# The relationship between self-reported immune fitness and salivary immune biomarker concentrations

**DOI:** 10.1007/s12026-025-09654-1

**Published:** 2025-06-12

**Authors:** Emina Išerić, Pauline A Hendriksen, Guusje A Ulijn, Marit Zuurveld, Aurora JAE van de Loo, Johan Garssen, Joris C. Verster

**Affiliations:** 1 Division of Pharmacology, Utrecht University, Utrecht Institute for Pharmaceutical Sciences, Universiteitsweg 99, Utrecht, 3584 CG the Netherlands; 2 Danone Global Research and Innovation Center, Uppsalalaan 12, Utrecht, 3584CT the Netherlands; 3 Cognitive Neurophysiology, Department of Child and Adolescent Psychiatry, Faculty of Medicine, TU Dresden, Dresden, 01307 Germany; 4 Centre for Mental Health and Brain Sciences, Swinburne University, Melbourne, VIC 3122 Australia

**Keywords:** Immune fitness, Immunity, Self-report, Biomarkers, Saliva

## Abstract

Immune fitness refers to the body’s ability to respond to health challenges by activating an appropriate immune response. Perceived immune fitness can be assessed using a single-item scale ranging from 0 (very poor) to 10 (excellent). The aim of the current study (n = 29 healthy volunteers) was to evaluate the relationship between perceived immune fitness and immune biomarker concentrations in saliva. Hourly assessments of immune fitness were made throughout the day (09:30 – 15:30), and saliva samples were collected accordingly. The concentrations of interleukin (IL)-1β, IL-6, IL-8, and tumor necrosis factor-alpha (TNF-α) were determined using multiplex immunoassay. While immune fitness scores remained stable during the day, biomarker assessments showed some fluctuations. For IL-6, significant negative correlations were found between IL-6 concentration at 10:30 and immune fitness scores at 10:30 (r = -0.512), 11:30 (r = -0.383), and 12:30 (r = -0.443), and between the IL-6 concentration and immune fitness score at 15:30 (r = -0.704). For IL-8, significant correlations were found between IL-8 concentration at 10:30 and immune fitness scores at 10:30 (r = -0.480), 12:30 (r = -0.456), and 14:30 (r = -0.429). For TNF-α, significant positive correlations were found between TNF-α concentration at 13:30 and immune fitness scores at 13:30 (r = 0.517) and 14:30 (r = 0.477). No significant correlations were found between immune fitness and IL-1β. In conclusion, immune fitness scores remained stable throughout the day, and were significantly associated with salivary concentrations of IL-6, IL-8, and TNF-α at certain time points.

## Introduction

Immune fitness refers to the body’s ability to respond to health challenges by activating an appropriate immune response [1]. Adequate immune fitness is crucial for maintaining health, and reduced immune fitness has been associated with various chronic immune-related diseases, such as diabetes and cardiovascular disease [2–4], as well as with minor immune-related complaints [5]. Immune fitness reflects an individual’s overall perception of how well their immune system functions. Various factors determine immune fitness, including the type, severity, duration, and frequency of immune-related complaint(s), as well as the individual’s ability to cope with these complaints and the extent to which they impact daily functioning [1, 6].

Immune fitness can be assessed via an 11-point single-item scale, ranging from 0 (very poor) to 10 (excellent) [1]. The single-item immune fitness scale demonstrated excellent test–retest reliability [7]. Although the risk of immune-related diseases may vary across seasons – for example, with the occurrence of common colds or allergies – previous research has shown that immune fitness scores in healthy individuals remained stable throughout the year [8].

In healthy individuals, self-assessed immune fitness scores typically range from 6 to 10, with lower scores usually indicating reduced immune fitness. When immune fitness is adequate, concentrations of immune biomarkers are expected to remain within the normal range. However, in cases of reduced immune fitness – for example, due to acute illnesses such as the common cold, or in the presence of an underlying disease – changes in biomarker concentrations may occur.

Despite its relevance, research examining the relationship between single-item immune fitness scores and objective immune biomarker assessments is limited. Mulder et al. [9] found no significant correlations between single-item immune fitness scores and salivary concentrations of C-reactive protein (CRP), interleukin (IL)−1β, IL-8, and immunoglobulin A (IgA) in n = 108 healthy young adults. Concentrations of IL-6, IL-10, and tumor necrosis factor alpha (TNF-α) were below the limit of detection and could therefore not be analyzed. To date, no other studies have been published that examined associations between single-item immune fitness scores and immune biomarker outcomes.

Therefore, the aim of the current study was to further evaluate the extent to which perceived immune fitness and immune biomarker concentrations are related. Hourly assessments were conducted throughout the day (09:30–15:30). Biomarkers were assessed in saliva, as previous research has shown that salivary concentrations correlate with those in blood [10]. Moreover, clinical trial participants generally prefer non-invasive procedures (e.g., saliva collection) over invasive methods such as a blood sampling [11]. This is particularly relevant in the current study, which involved seven data collection time points. It was hypothesized that significant correlations would be found between immune fitness scores and biomarker concentrations. However, given that healthy volunteers participated, it was also expected that the variability in outcomes would be relatively limited (i.e., within the normal range), resulting in modest correlations.

## Methods

Data from the control day (no intervention, no alcohol consumption on the previous day) of a study evaluating alcohol hangover effects were used for the current analysis [12]. The study was approved by the Psychology Ethics Committee of the University of Groningen (protocol code: ppo-015–002, approval date: 3 September 2015). Healthy volunteers (social drinkers) were recruited via local advertisement, and written informed consent was obtained prior to participation. Participants received €120,—for completing the study.

Inclusion criteria were being a healthy male or female, aged between 18 and 30 years. Exclusion criteria at screening included smoking, drug use, use of medication, or the presence of any underlying disease. On the test day, participants were further excluded if they experienced minor (immune-related) illness or reported poor sleep quality during the preceding night.

At the start of the test day, a medical examination was conducted by the study physician to confirm the participants’ health status and medical history. The use of illicit drugs (including amphetamines, barbiturates, cannabinoids, benzodiazepines, cocaine, and opiates) was verified via a urine drug test (AlfaScientic Designs Inc., Poway, CA, USA). Recent alcohol consumption was verified using the Alcotest 7410 Breath Alcoholmeter (Dräger, Hoogvliet, the Netherlands), ensuring a breath alcohol concentration of 0,00%.

The test day involved hourly assessments (09:30–15:30) of immune fitness and saliva collection for biomarker analysis. Between assessments, participants were instructed to relax (e.g., read a book). No food or drinks were allowed, except for a standardized breakfast at 09:00 and a standardized lunch at 12:00. Moderate, standardized amounts of water were provided if needed.

Immune fitness was assessed hourly (09:30—15:30) using a 10-cm visual analog scale (VAS), ranging from 0 (very poor) to 10 (excellent). Participants indicated their immune fitness score by placing and ‘X’ on the VAS, which was then measured in cm, with higher scores reflecting better immune fitness.

Saliva samples were collected hourly (09:30—15:30) using a passive drool method (SalivaBio’s Saliva Collection Aid, Salimetrics, State College, PA, USA). Samples were immediately stored at −80 degrees Celsius after collection.

Salivary concentrations (pg/ml) of IL-1β, IL-2, IL-4, IL-5, IL-6, IL-8, IL-10, granulocyte–macrophage colony-stimulating factor (GM-CSF), interferon-gamma (IFN-γ) and TNF-α were determined using a multiplex immunoassay (customized ProcartaPlex Immunoassay, ThermoFisher Scientific, Waltham, USA), following the manufacturer’s instructions and standard procedures described elsewhere [13].

For each multiplex plate, the lower limit of detection (LOD) was computed. For biomarker concentrations below the LOD, a value corresponding to half the LOD was used in the statistical analyses. Biomarkers with > 25% of assessments below LOD (i.e., IL-2, IL-4, IL-5, IL-10, GM-CSF, and IFN-α) were considered unreliable and excluded from further analyses.

All statistical analyses were conducted using SPSS (IBM Corp. Released 2013. IBM SPSS Statistics for Windows, Version 30. Armonk, NY, USA: IBM Corp.). The distribution of the data was evaluated via visual inspection and the Kolmogorov–Smirnov test and revealed that not all data was normally distributed. Immune fitness and biomarker concentrations (IL-1β, IL-6, IL-8, and TNF-α) assessed throughout the day (09:30–15:30) were compared to the morning assessment (09:30) using the Independent-Samples Kruskal–Wallis test. After applying Bonferroni’s correction for multiple comparisons, differences were considered significant if *p* < 0.0083. 

Spearman’s correlations were computed to examine associations between immune fitness and biomarker concentrations at different time points. To account for the relatively small sample size, bootstrapping was applied (B = 10,000 samples), and bias-corrected and accelerated 95% confidence intervals (BCa 95% CI_B_) were calculated [14, 15]. The BCa 95% CI_B_ ranges from − 1 to + 1, with narrower intervals indicating greater precision. Correlations were considered statistically significant if the BCa 95% CI_B_ did not contain zero.

## Results

**Fig. 1:**
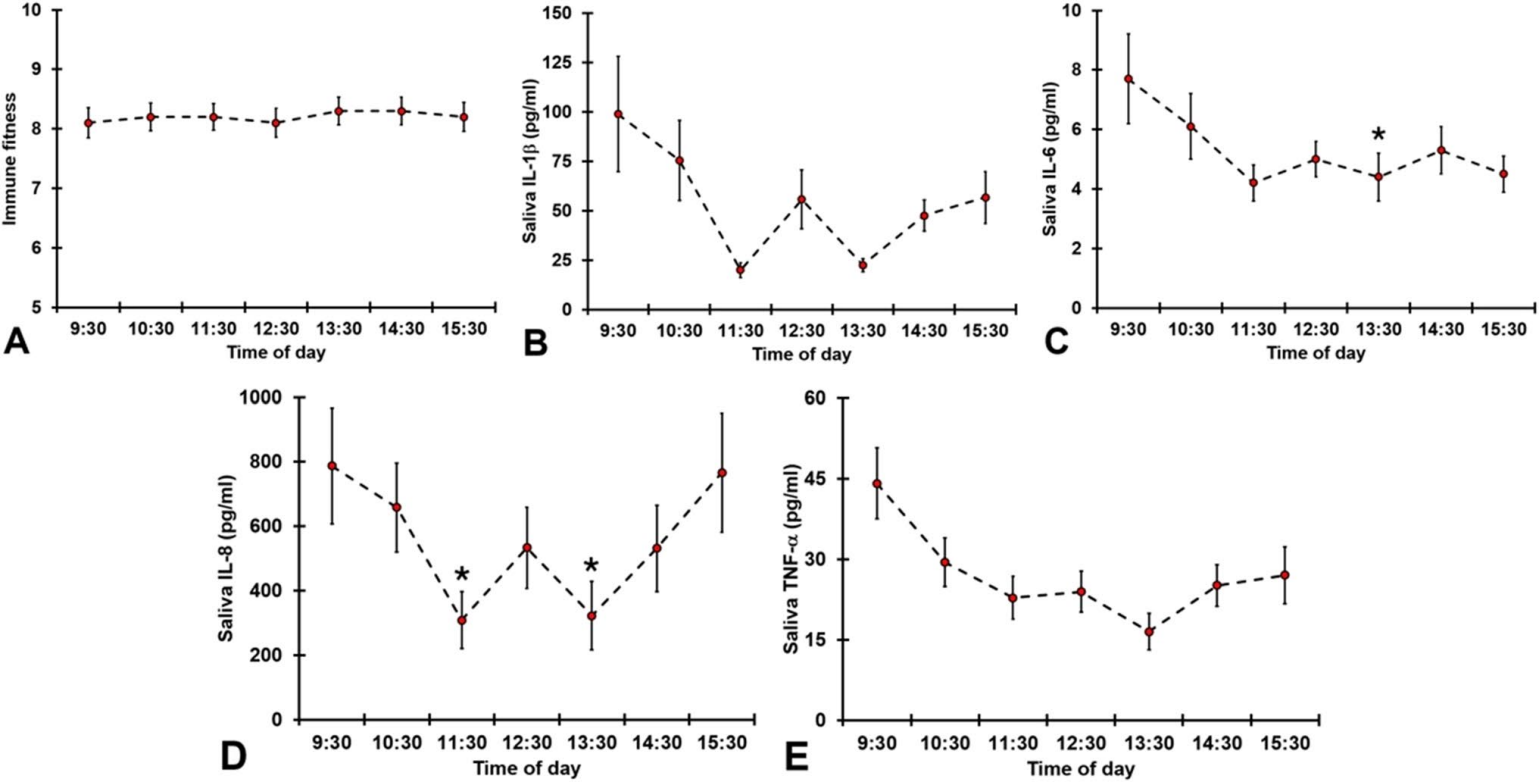
Immune fitness and salivary biomarker concentrations throughout the day. Mean values and standard errors (SE) are shown for each time point for (a) immune fitness, (b) IL-1β, (c) IL-6, (d) IL-8, and (e) TNF-α. Significant differences compared to the 09:30 assessment (p < 0.0083, Bonferroni-corrected) are indicated by an asterisk (*). Abbreviations: IL = interleukin, TNF-α = tumor necrosis factor-alpha

A total of 29 participants (15 males, 14 females) participated in the study. Their mean age was 21.1 years (SD = 2.0). Figure 1 displays the hourly immune fitness scores and salivary biomarker concentrations across the day.

No significant variation over time was observed for immune fitness (p = 0.572; see Fig. 1a). Significant time effects were found for IL-1β (p = 0.003), with lower concentrations observed in the afternoon (Fig. 1b). However, compared to the 09:30 assessment, no significant pairwise differences were found for IL-1β at any later time points. For IL-6, a significant time effect was observed (p = 0.032), with lower concentrations in the afternoon (Fig. 1c). Specifically, IL-6 levels at 13:30 were significantly lower than those at 09:30 (p = 0.008); other time points did not differ significantly.

IL-8 also showed a significant change over time (p = 0.008), with lower concentrations in the afternoon (Fig. 1d). Compared to 09:30, IL-8 concentrations were significantly lower at 11:30 (p = 0.003) and 13:30 (p = 0.002). Although no overall significant time effect was found for TNF-α (p = 0.161), lower TNF-α concentrations were observed in the afternoon (Fig. 1e).

Spearman’s correlations were computed to examine associations between immune fitness scores and salivary biomarker concentrations, and were confirmed using bootstrapping analysis. The results are summarized in Tables 1 – 4.

**Table 1:** Correlations between immune fitness and salivary IL-1β concentrations

Time (h)	Time of immune fitness assessment						
	09:30	10:30	11:30	12:30	13:30	14:30	15:30
**IL-1β (09:30)**							
r	−0.311	−0.238	−0.210	−0.262	−0.293	−0.206	−0.303
p-value	0.114	0.233	0.293	0.187	0.138	0.304	0.125
BCa 95% CI_B LOWER_	−0.661	−0.594	−0.563	−0.630	−0.638	−0.596	−0.667
BCa 95% CI_B HIGHER_	0.091	0.167	0.194	0.167	0.129	0.227	0.123
**Il-1β (10:30)**							
r	X	−0.381	−0.241	−0.312	−0.267	−0.326	−0.298
p-value	X	0.061	0.245	0.128	0.196	0.112	0.148
BCa 95% CI_B LOWER_	X	−0.735	−0.646	−0.695	−0.660	−0.718	−0.683
BCa 95% CI_B HIGHER_	X	0.093	0.264	0.183	0.236	0.188	0.173
**IL-1β (11:30)**							
r	X	X	−0.205	−0.030	−0.082	−0.038	−0.037
p-value	X	X	0.361	0.895	0.715	0.865	0.875
BCa 95% CI_B LOWER_	X	X	−0.668	−0.558	−0.592	−0.538	−0.588
BCa 95% CI_B HIGHER_	X	X	0.320	0.478	0.448	0.459	0.501
**IL-1β (12:30)**							
r	X	X	X	0.201	0.180	0.112	0.243
p-value	X	X	X	0.347	0.401	0.601	0.253
BCa 95% CI_B LOWER_	X	X	X	−0.174	−0.213	−0.315	−0.153
BCa 95% CI_B HIGHER_	X	X	X	0.526	0.543	0.493	0.579
**IL-1β (13:30)**							
R	X	X	X	X	0.086	−0.017	−0.108
p-value	X	X	X	X	0.683	0.937	0.607
BCa 95% CI_B LOWER_	X	X	X	X	−0.321	−0.418	−0.501
BCa 95% CI_B HIGHER_	X	X	X	X	0.474	0.381	0.307
**IL-1β (14:30)**							
r	X	X	X	X	X	−0.147	−0.156
p-value	X	X	X	X	X	0.464	0.437
BCa 95% CI_B LOWER_	X	X	X	X	X	−0.528	−0.599
BCa 95% CI_B HIGHER_	X	X	X	X	X	0.269	0.275
**IL-1β (15:30)**							
r	X	X	X	X	X	X	−0.390
p-value	X	X	X	X	X	X	0.060
BCa 95% CI_B LOWER_	X	X	X	X	X	X	−0.723
BCa 95% CI_B HIGHER_	X	X	X	X	X	X	0.053

Spearman’s correlations were computed between salivary IL-1β concentrations and immune fitness scores at each time point. Bootstrapping (B = 10.000 samples) was applied, and bias-corrected and accelerated 95% confidence intervals (BCa 95%CI_B_) were calculated. A bootstrap correlation was considered significant if the BCa 95%CI_B_ did not include zero. X = no assessment

No significant correlations were found between immune fitness and IL-1β (Table 1). For IL-6 (Table 2), significant negative correlations were observed between IL-6 concentration at 10:30 and immune fitness scores at 10:30 (r = −0.512), 11:30 (r = −0.383), and 12:30 (r = −0.443). Additionally, a significant negative correlation was found between IL-6 concentration at 15:30 and immune fitness score at 15:30 (r = −0.704).

**Table 2:** Correlations between immune fitness and salivary IL-6 concentrations

Time (h)	Time of immune fitness assessment						
	09:30	10:30	11:30	12:30	13:30	14:30	15:30
**IL-6 (09:30)**							
r	−0.182	−0.191	−0.176	−0.192	−0.223	0.010	−0.248
p-value	0.364	0.340	0.379	0.337	0.263	0.961	0.213
BCa 95% CI_B LOWER_	−0.562	−0.537	−0.502	−0.530	−0.526	−0.363	−0.570
BCa 95% CI_B HIGHER_	0.222	0.187	0.172	0.176	0.115	0.372	0.091
**Il-6 (10:30)**							
r	X	**−0.512**	**−0.383**	**−0.443**	−0.354	−0.321	−0.338
p-value	X	**0.006**	**0.048**	**0.021**	0.070	0.102	0.085
BCa 95% CI_B LOWER_	X	**−0.742**	**−0.665**	**−0.703**	−0.639	−0.625	−0.660
BCa 95% CI_B HIGHER_	X	**−0.166**	**−0.004**	**−0.086**	0.022	0.058	0.057
**IL-6 (11:30)**							
r	X	X	−0.177	−0.149	−0.108	−0.105	−0.133
p-value	X	X	0.387	0.469	0.603	0.608	0.518
BCa 95% CI_B LOWER_	X	X	−0.532	−0.513	−0.487	−0.472	−0.531
BCa 95% CI_B HIGHER_	X	X	0.221	0.246	0.302	0.298	0.298
**IL-6 (12:30)**							
r	X	X	X	−0.034	0.130	−0.095	−0.014
p-value	X	X	X	0.872	0.536	0.652	0.948
BCa 95% CI_B LOWER_	X	X	X	−0.424	−0.269	−0.477	−0.411
BCa 95% CI_B HIGHER_	X	X	X	0.367	0.504	0.325	0.386
**IL-6 (13:30)**							
r	X	X	X	X	0.260	0.240	0.170
p-value	X	X	X	X	0.190	0.227	0.397
BCa 95% CI_B LOWER_	X	X	X	X	−0.052	−0.101	−0.199
BCa 95% CI_B HIGHER_	X	X	X	X	0.534	0.523	0.506
**IL-6 (14:30)**							
r	X	X	X	X	X	0.006	−0.190
p-value	X	X	X	X	X	0.976	0.341
BCa 95% CI_B LOWER_	X	X	X	X	X	−0.373	−0.593
BCa 95% CI_B HIGHER_	X	X	X	X	X	0.374	0.231
**IL-6 (15:30)**							
r	X	X	X	X	X	X	**−0.704**
p-value	X	X	X	X	X	X	** < 0.001**
BCa 95% CI_B LOWER_	X	X	X	X	X	X	**−0.904**
BCa 95% CI_B HIGHER_	X	X	X	X	X	X	**−0.339**

Spearman’s correlations were computed between salivary IL-6 concentrations and immune fitness at each time point. Bootstrapping (B = 10.000 samples) was applied, and bias-corrected and accelerated 95% confidence intervals (BCa 95%CI_B_) were calculated. A bootstrap correlation was considered significant if the BCa 95%CI_B_ did not include zero. Significant correlations are shown in bold. X = no assessment

For IL-8 (Table 3), significant negative correlations were found between IL-8 concentration at 10:30 and immune fitness scores at 10:30 (r = −0.480), 12:30 (r = −0.456), and 14:30 (r = −0.429).

**Table 3:** Correlations between immune fitness and salivary IL-8 concentrations

Time (h)	Time of immune fitness assessment						
	09:30	10:30	11:30	12:30	13:30	14:30	15:30
**IL-8 (09:30)**							
r	−0.129	−0.156	−0.096	−0.184	−0.171	−0.122	−0.209
p-value	0.548	0.466	0.654	0.389	0.424	0.569	0.326
BCa 95% CI_B LOWER_	−0.536	−0.574	−0.491	−0.592	−0.532	−0.561	−0.587
BCa 95% CI_B HIGHER_	0.309	0.302	0.303	0.292	0.224	0.379	0.233
**Il-8 (10:30)**							
r	X	**−0.480**	−0.418	**−0.456**	−0.373	**−0.429**	−0.323
p-value	X	**0.010**	0.027	**0.015**	0.051	**0.023**	0.093
BCa 95% CI_B LOWER_	X	**−0.753**	−0.716	**−0.730**	−0.678	**−0.726**	−0.636
BCa 95% CI_B HIGHER_	X	**−0.077**	0.004	**−0.048**	0.039	**−0.013**	0.092
**IL-8 (11:30)**							
r	X	X	−0.094	−0.054	−0.040	−0.095	0.049
p-value	X	X	0.646	0.793	0.846	0.645	0.814
BCa 95% CI_B LOWER_	X	X	−0.512	−0.478	−0.458	−0.495	−0.376
BCa 95% CI_B HIGHER_	X	X	0.356	0.406	0.410	0.369	0.456
**IL-8 (12:30)**							
r	X	X	X	−0.235	−0.204	−0.293	−0.078
p-value	X	X	X	0.249	0.318	0.146	0.705
BCa 95% CI_B LOWER_	X	X	X	−0.598	−0.572	−0.643	−0.536
BCa 95% CI_B HIGHER_	X	X	X	0.192	0.203	0.126	0.376
**IL-8 (13:30)**							
r	X	X	X	X	0.005	−0.097	−0.008
p-value	X	X	X	X	0.981	0.637	0.970
BCa 95% CI_B LOWER_	X	X	X	X	−0.413	−0.501	−0.433
BCa 95% CI_B HIGHER_	X	X	X	X	0.449	0.370	0.422
**IL-8 (14:30)**							
r	X	X	X	X	X	−0.100	−0.151
p-value	X	X	X	X	X	0.626	0.463
BCa 95% CI_B LOWER_	X	X	X	X	X	−0.518	−0.570
BCa 95% CI_B HIGHER_	X	X	X	X	X	0.380	0.311
**IL-8 (15:30)**							
r	X	X	X	X	X	X	−0.155
p-value	X	X	X	X	X	X	0.450
BCa 95% CI_B LOWER_	X	X	X	X	X	X	−0.570
BCa 95% CI_B HIGHER_	X	X	X	X	X	X	0.280

Spearman’s correlations were computed between salivary IL-8 concentrations and immune fitness at each time point. Bootstrapping (B = 10.000 samples) was applied, and bias-corrected and accelerated 95% confidence intervals (BCa 95%CI_B_) were calculated. A bootstrap correlation was considered significant if the BCa 95%CI_B_ did not include zero. Significant correlations are shown in bold. X = no assessment

For TNF-α (Table 4), significant positive correlations were found between TNF-α concentration at 13:30 and immune fitness scores at 13:30 (r = 0.517) and 14:30 (r = 0.477).

**Table 4:** Correlations between immune fitness and salivary TNF-α concentrations

Time (h)	Time of immune fitness assessment						
	09:30	10:30	11:30	12:30	13:30	14:30	15:30
**TNF-α (09:30)**							
r	−0.160	−0.123	−0.130	−0.122	−0.187	0.047	−0.203
p-value	0.415	0.532	0.511	0.535	0.342	0.812	0.300
BCa 95% CI_B LOWER_	−0.542	−0.502	−0.497	−0.510	−0.524	−0.353	−0.572
BCa 95% CI_B HIGHER_	0.291	0.299	0.276	0.291	0.212	0.435	0.221
**TNF-α (10:30)**							
r	X	−0.408	−0.197	−0.338	−0.216	−0.212	−0.370
p-value	X	0.039	0.334	0.092	0.289	0.297	0.063
BCa 95% CI_B LOWER_	X	−0.699	−0.554	−0.656	−0.576	−0.560	−0.675
BCa 95% CI_B HIGHER_	X	0.007	0.219	0.074	0.209	0.218	0.046
**TNF-α (11:30)**							
r	X	X	0.084	0.109	0.063	−0.001	0.083
p-value	X	X	0.682	0.595	0.761	0.995	0.688
BCa 95% CI_B LOWER_	X	X	−0.365	−0.336	−0.340	−0.426	−0.307
BCa 95% CI_B HIGHER_	X	X	0.506	0.529	0.477	0.414	0.473
**TNF-α (12:30)**							
r	X	X	X	0.216	0.323	0.222	0.214
p-value	X	X	X	0.289	0.108	0.275	0.293
BCa 95% CI_B LOWER_	X	X	X	−0.167	−0.058	−0.178	−0.167
BCa 95% CI_B HIGHER_	X	X	X	0.541	0.637	0.555	0.556
**TNF-α (13:30)**							
r	X	X	X	X	**0.517**	**0.477**	0.376
p-value	X	X	X	X	**0.006**	**0.012**	0.053
BCa 95% CI_B LOWER_	X	X	X	X	**0.209**	**0.165**	−0.006
BCa 95% CI_B HIGHER_	X	X	X	X	**0.760**	**0.727**	0.683
**TNF-α (14:30)**							
r	X	X	X	X	X	0.028	−0.115
p-value	X	X	X	X	X	0.892	0.568
BCa 95% CI_B LOWER_	X	X	X	X	X	−0.373	−0.509
BCa 95% CI_B HIGHER_	X	X	X	X	X	0.402	0.279
**TNF-α (15:30)**							
r	X	X	X	X	X	X	−0.245
p-value	X	X	X	X	X	X	0.239
BCa 95% CI_B LOWER_	X	X	X	X	X	X	−0.633
BCa 95% CI_B HIGHER_	X	X	X	X	X	X	0.219

Spearman’s correlations were computed between salivary TNF-α concentrations and immune fitness at each time point. Bootstrapping (B = 10.000 samples) was applied, and bias-corrected and accelerated 95% confidence intervals (BCa 95%CI_B_) were calculated. A bootstrap correlation was considered significant if the BCa 95%CI_B_ did not include zero. Significant correlations are shown in bold. X = no assessment

## Discussion

This study found that while immune fitness scores remained stable throughout the day, salivary concentrations of several biomarkers—specifically IL-6 and IL-8—showed significant diurnal variation. The within-day variability for IL-1β and TNF-α did not reach statistical significance. Significant correlations were observed between immune fitness and IL-6 and IL-8 during some of the morning assessments, and between immune fitness and TNF-α during some of the afternoon assessments. No significant correlations were found between immune fitness scores and IL-1β concentrations.

These findings align with previous studies that reported diurnal variability in salivary immune biomarkers. For example, research in healthy toddlers showed contrasting diurnal patterns for alpha-amylase immunoglobulin A (IgA) [16]. However, in adults, cytokine levels measured in serum or blood are generally considered not to exhibit strong circadian rhythms [17]. Still, some variation across the day was observed in the present study.

In a study among severe trauma patients, circulating levels of T lymphocytes and monocytes peaked during the night, while neutrophils counts were highest during the day [18]. As monocytes and T lymphocytes are major producers of IL-1β, IL-6, IL-8 and TNF-α, the modest diurnal variations observed here in saliva may reflect corresponding fluctuations in these immune cell populations.

Finally, although some of the observed differences in salivary biomarker concentrations reached statistical significance, their clinical relevance may be limited. The magnitude of these fluctuations was relatively small, and the observed concentrations were consistent with those previously reported in healthy, young social drinkers [13].

The cytokines IL-1β, IL-6, IL-8 and TNF-α are generally considered proinflammatory mediators, promoting inflammatory responses [19]. Accordingly, the observed negative correlations between immune fitness and IL-6 and IL-8 concentrations align with their well-established proinflammatory roles.

Despite its proinflammatory nature, TNF-α showed a positive correlation with immune fitness in the present study. Although this may appear counterintuitive, TNF-α is also known to exert anti-inflammatory functions. For example, it can suppress the release of proinflammatory cytokines from murine macrophages [20], and promote the secretion of regulatory mediators such as IL-10 from human macrophages via mesenchymal stem cells [21].

These varying correlations between salivary cytokine concentrations and immune fitness may therefore reflect the complex regulatory mechanisms through which the immune system maintains homeostasis.

The significant correlations observed were of moderate to good magnitude (0.383 < r < 0.704). However, correlations did not reach statistical significance at most time points. This is understandable, given that only healthy volunteers participated in the study. Consequently, both biomarker concentrations and immune fitness scores were largely within the normal range, resulting in relatively small between-subject variability.

It is important to note that immune fitness reflects an individual’s overall perception of how well their immune system functions, including the type, severity, duration, and impact of any immune-related complaints experienced [1]. In contrast, cytokine biomarkers primarily indicate the presence or absence of inflammation. Moreover, the immune response is highly complex and regulate by over 150 cytokines [22], which interact to orchestrate immune activity [23]. Therefore, the assessment of a single cytokine is unlikely to capture the overall functioning of the immune system (i.e., immune fitness). This likely explains why, at best, only modest correlations were found between cytokine concentrations and immune fitness scores.

This study is one of the first to demonstrate that perceived immune fitness scores and objective immune biomarker concentrations are related. A key strength of the study is the repeated assessment of both immune fitness and biomarkers at 7 time points throughout the day. In addition, participants were carefully selected healthy volunteers who were thoroughly screened by a study physician.

A limitation of the study is its relatively small sample size. While the study was sufficiently powered for the original hangover trial, a larger sample size would be preferable for correlational analyses performed here. To address this, bootstrapping was applied to account for the limited sample size. Other limitations include the narrow age range of participants (18–30 years), and the absence of systematic assessment of lifestyle factors that could influence immune fitness, such as physical activity or daily diet. However, it could be argued that such factors, if relevant, would likely have a similar impact on both immune fitness scores and biomarker concentrations.

Taken together, future studies with larger and more diverse samples – including broader age ranges and participants with underlying health conditions – are warranted to replicate and confirm the current findings.

In conclusion, this study found significant correlations between single-item immune fitness scores and salivary immune biomarker concentrations at specific time points. Further research in larger and more diverse samples is warranted to replicate and extend these findings. 

## Data Availability

The data is available from the corresponding author upon reasonable request.
